# Metastatic Urachal Carcinoma Treated With Several Different Combined Regimens: A Case Report

**DOI:** 10.3389/fonc.2021.662589

**Published:** 2021-07-16

**Authors:** Han Zheng, Wei Song, Xiemin Feng, Hong Zhao

**Affiliations:** Department of Medical Oncology, The First Affiliated Hospital of Yan’an University, Yan’an, China

**Keywords:** urachal carcinoma, serum marker, immunity therapy, targeted therapy, combination chemotherapy

## Abstract

Urachal carcinoma is a rare bladder malignance. This study presents a case of an elderly patient with urachal carcinoma who was found to have pulmonary metastases 1 year after 5 recurrent resections. The patient was treated with up to 7 different chemotherapy regimens, including a VEGF monoclonal antibody and anti-PD-1 antibody. This is the first report of PD-1 antibody being used in patients with urachus, although the disease progressed after only four cycles of the application. The patient’s disease was controlled by the FOLFIRI combined with the VEGF monoclonal antibody regimen. The most prominent issues at present are the difficulty of obtaining drugs for rare cancers and the lack of late-stage clinical trials to guide therapeutic decisions.

## Introduction

The first case of urachal carcinoma was reported by Begg in 1973 (1, 2), and subsequent reports are based on single case descriptions and institutional experiences. The urachus, a fibrous remnant of the allantois, usually regresses during fetal life, but its lumen stays in place in approximately one-third of adults. Urachal carcinoma accounts for 0.35%–0.7% of all bladder tumors and presents at an advanced stage and poor prognosis. For different types of pathology, there are different surgical approaches, among which, immunotherapy and targeted therapies for urachal carcinoma are still being explored.

## Case Presentation

**Figure 1:**
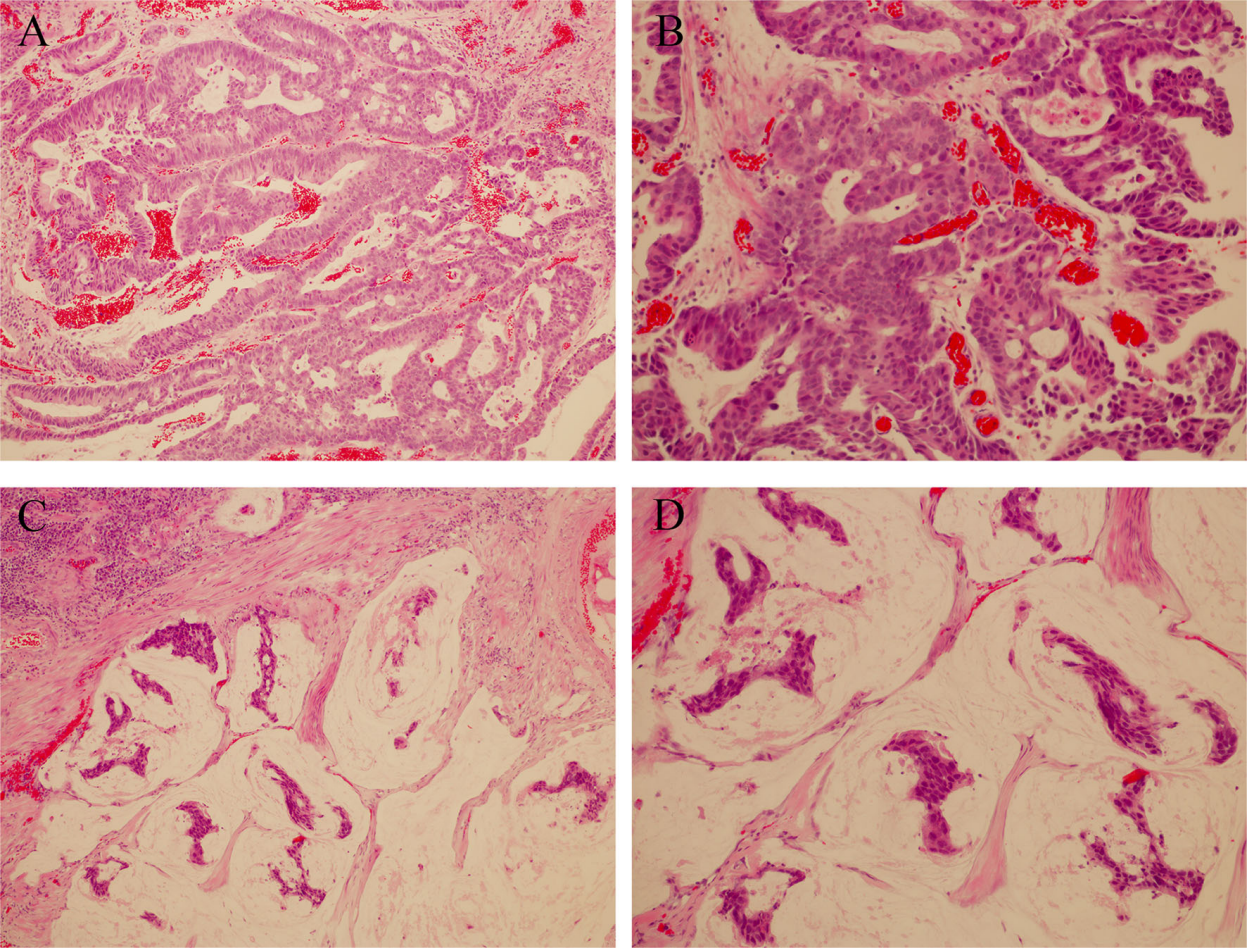
**(A, B)** Hematoxylin and eosin staining; magnification ×100 and ×200, Duct cells are observed in the loose stroma. **(C, D)** Hematoxylin and eosin staining; magnification ×100 and ×200, Urachal mucinous adenocarcinoma, groups of tumour cells surrounded by extracellular mucin.

**Figure 2:**
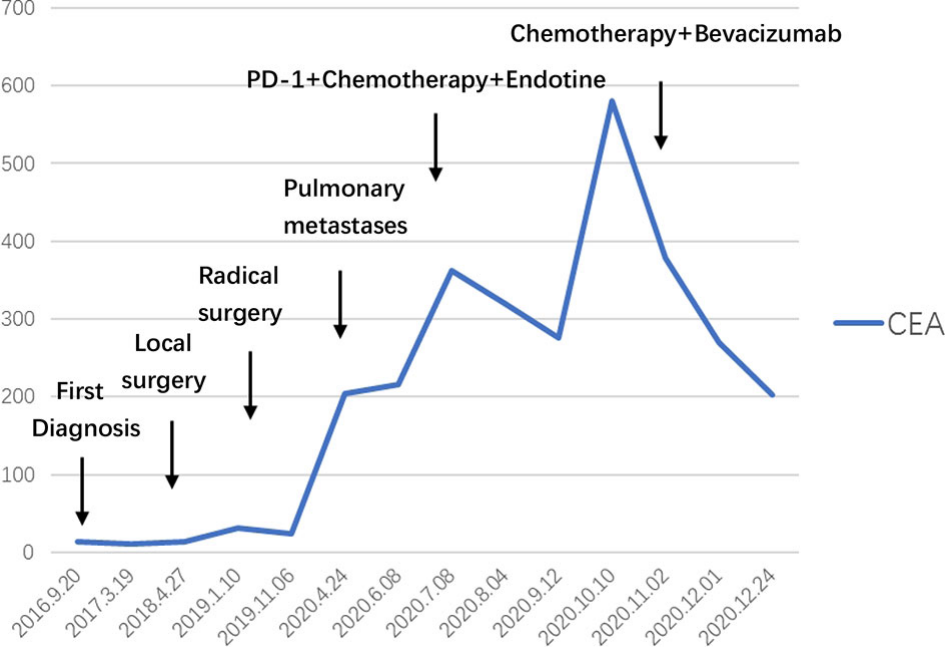
Timeline of the Serum marker.

Patient: a 62-year-old male presented with a history of gross hematuria for 1 month. The patient immediately underwent a transurethral biopsy of the urachal tumor, which revealed a 3.0×2.0×3.0 cm adenocarcinoma and some mucinous adenocarcinoma (**Figure 1**). He requested to preserve the bladder to ensure the quality of life. When mucinous adenocarcinoma was again found in the bladder 3 months later, he began to receive systemic treatment with a standard bladder carcinoma regimen consisting of gemcitabine and cisplatin, which lasted for 4 cycles and followed by a 2 transurethral tumour electrosurgery within 3 years. He finally underwent a radical cystectomy 3 years after the onset of the disease. Pathology showed a 6.0×5.3 cm adenocarcinoma with positive immunohistochemistry for AE1/AE3, CK7, CK20, and CK19. Four weeks after surgery with paclitaxel and cisplatin, the patient began to receive chemotherapy, which was repeated every 3 weeks for 4 cycles. CT scans that performed after a treatment of 8 months showed progressive pulmonary metastases. The patient started to receive treatment with gemcitabine and nedaplatin in combination with Endostar. A CT scan performed after a treatment of 3 cycles (3 months) showed no significant reduction in the lesion, so anti-PD-1 antibody was added. This regimen was well-tolerated, and CT scans done after 3 cycles showed stable disease. However, scans after 5 cycles showed significant clinical and radiological progression, after which, he was discontinued from the anti-PD-1 antibody and switched to bevacizumab, irinotecan, 5-fluorouracil (5-FU), and folinic acid, which are delivered weekly for 3 weeks **(** **Table 1** **)**. After 6 cycles, a 50% reduction in the size of the metastatic lung lesions was observed by CT and the CEA decreased from 588.1 to 205.7 ng/ml (**Figure 2**).

**Table 1:** Details about the treatments (schedule and dosage).

Time	Programme		CEA ng/ml
2015.11	DDP 40 mg, GEM 1.8 g	4 cycle	19.2
2019.03	DDP 30 mg, Taxol 400 mg	4 cycle	17.3
2020.04	NDP 120 mg, GEM 1800 mg, YH-16 30 mg	3 cycle	307.9
2020.07	NDP 120 mg, GEM 1800 mg, Tislelizumab 200 mg	3 cycle	588.1
2020/10	Irinotecan 280 mg, CF 750 mg, 5-FU 4.9 g Bevacizumab 500 mg	6 cycle	205.7

## Discussion

Urachal carcinoma shares certain histological and biological characteristics with intestinal tumour, and there are no clinical randomised trials available for its treatement due to its rarity. Early symptoms of both urachal and bladder carcinoma are gross hematuria visible to the naked eye, so they are often confused. However, patients with urachal carcinoma often have mucus-like substance in their urine, which is consistent with its type of pathology (3). In 2006, Asley et al. (4) proposed the Mayo staging, which is simpler and clearer than any previous staging. Initially, the overall prognosis for urachal carcinoma was low, with a 5-year survival rate of only 6% (5), but the rate has been improving with the use of new drugs. As with other intestinal malignancies, the persistent elevation of tumour markers predicts disease progression, with CEA, CA125, and CA19-9 markers being the most closely associated with the disease. Siefker-Radtke (6) found elevated serum CEA levels in 59% of patients with urachal carcinoma. Zong (7) noted that the elevated CEA and CA125 are suggestive of the recurrence of urachal carcinoma. In our case, we observed that serum CEA significantly increased after pulmonary metastases. After treatment, the decrease in marker levels may be correlated with the response to systemic therapy.

Surgery is the main form of treatment for urachal carcinoma. En bloc surgical removal of the umbilicus, urachal ligament, and partial cystectomy with pelvic lymphadenectomy are the preferred interventions (8). However, unlike other carcinomas, no standard adjuvant chemotherapy regimen is yet available for treatment. In this reported case, the patient underwent five successive surgeries, and he was not immune to distant metastases despite the use of postoperative adjuvant chemotherapy. The choice of regimens was based largely on case reports and single institution experiences, and we have seen better response rates with 5-FU based regimens. Szarvas et al. (9) found that two therapies combined (cisplatin-based and 5-FU-based) had the best response rate (43%) than being applied alone. Tazi E and Mohile SG each described patients’ response to chemotherapy with irinotecan, a commonly used regimen for colorectal carcinoma (10, 11). In our case, the patient’s markers continued to decline and the metastatic lesions shrank after the use of the FOLFIRI regimen.

**Figure 3:**
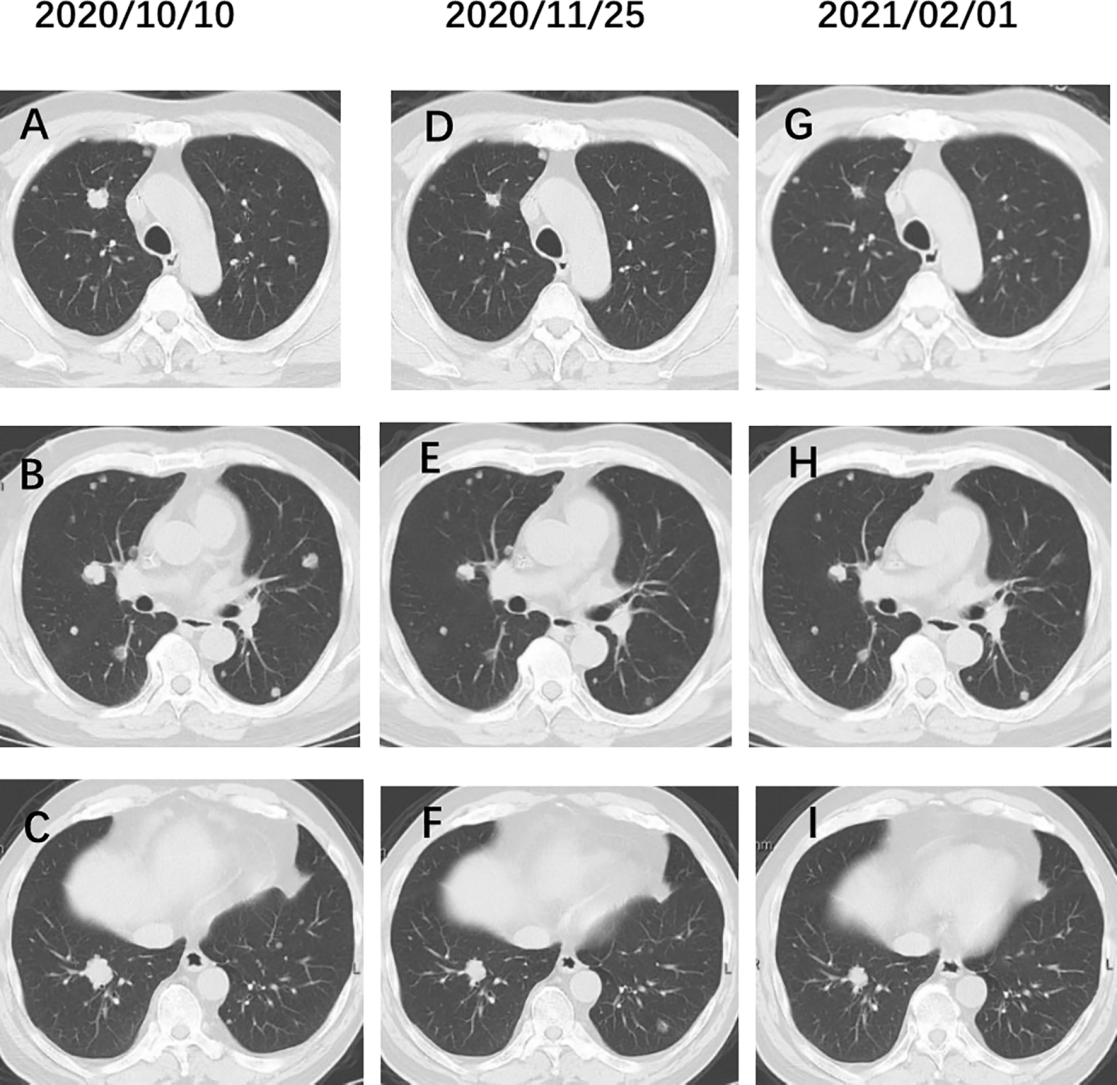
**(A–C)** Chest CT before administration of Irinotecan. **(D–F)** Chest CT after 3 cycles Irinotecan and bevacizumab therapy. **(G–I)** After 6 cycles Irinotecan and bevacizumab therapy. The pulmonary metastatic lesions were reduced by 50%.

In addition to the use of drugs targeting cytotoxicity, we used a combination of VEGF monoclonal antibody (bevacizumab) based on genetic testing that identified KRAS mutations in the patient (p.G12V), and achieved a favorable result of 50% reduction after 6 cycles Irinotecan and Bevacizumab therapy in metastatic lesions (**Figure 3**). Lee et al. (12) identified somatic SnVs/indels and SCANs in 17 patients by using the WES and OncoScan platforms. KRAS, MYC, and growth factors appear to be involved in pathogenesis, suggesting potential roles in targeted treatment of urachal cancer. Our case also confirms this result. KRAS mutations and microsatellite instability appear to be common in urachal carcinoma, and the presence of KRAS mutations is associated with better overall survival (OS) (13, 14). In intestinal tumours, we observed that patients with microsatellite instability do not benefit from 5-FU therapy, and similarly, it should be used with caution in patients with urachal carcinoma. Testa et al. (15) reported tumour regression in a patient with metastatic urachal carcinoma, who was treated with a second-line multikinase inhibitor (sunitinib) after failure of platinum-containing combination chemotherapy. Therefore, we suggest that mutation analysis targeting members of the EGFR pathway in patients with urachal carcinoma may provide additional therapeutic information.

Jordan Kardos’ study was the first overall transcriptome profiling of urachal carcinoma, demonstrating the similarity of this cancer to colorectal carcinoma (16). They reported tumors with mutations in DNA MMR proteins and POLE are rare, and described the successful treatment of a patient by using the anti-PD-L1 antibody atezolizumab. We treated the patient with an anti-PD-1 antibody, although it progressed after only 4 cycles, which is probably due to its low TMB, as mutational load correlates robustly with predicted neoantigen burden (17). The case reported by Sahu et al. was also treated with immunosuppressants, but the treatment was discontinued after one cycle due to immune pneumonia (18). By reviewing 76 articles (19), Claps concluded that personalized treatment could be the most suitable option for urachal carcinoma. Immunotherapy may have potential utility for urachal carcinoma, but clinical studies are still needed.

## Conclusion

Urachal carcinoma is highly similar to colorectal carcinoma, and the FOLFOX and FOLFIRI regimens for colorectal carcinoma are the most effective chemotherapy regimens for its treatment. We recommend performing mutational analysis for members of the EGFR pathway, as EGFR inhibitors are of interest for the treatment of urachal carcinoma. We look forward to the development of prospective clinical studies to further improve the diagnosis and treatment of ureteral carcinoma.

## Data Availability Statement

The original contributions presented in the study are included in the article/supplementary material. Further inquiries can be directed to the corresponding author.

## Ethics Statement

Written informed consent was obtained from the individual(s) for the publication of any potentially identifiable images or data included in this article.

## Author Contributions

HZhe and WS performed the research, wrote the paper. XF, HZhe, and WS were performed therapy to a patient. HZha (Correspondence) contributed to supervision of this study and revision of the manuscript. All authors contributed to the article and approved the submitted version.

## Funding

This study was supported by the Scientific Research Program of Education Department of Shaanxi Province (18JK0864).

## Conflict of Interest

The authors declare that the research was conducted in the absence of any commercial or financial relationships that could be construed as a potential conflict of interest.
